# The gut–brain axis: mechanisms linking intestinal dysbiosis with stroke

**DOI:** 10.3389/fcell.2026.1854765

**Published:** 2026-06-16

**Authors:** José Tomás Garrido Santos, Daniel Tornero, Marie Karam

**Affiliations:** 1 Laboratory of Neural Stem Cells and Brain Damage, Department of Biomedical Sciences, Institute of Neurosciences, University of Barcelona, Barcelona, Spain; 2 Institut d’Investigacions Biomèdiques August Pi i Sunyer (IDIBAPS), Barcelona, Spain; 3 Centro de Investigación Biomédica en Red Sobre Enfermedades Neurodegenerativas (CIBERNED), Madrid, Spain

**Keywords:** border associated macrophages, diet, ischemic stroke, microbiota, neuroinflammation

## Abstract

Gut microbiota has emerged as a key regulator of immune, metabolic, and neuroinflammatory processes, exerting significant influence on central nervous system (CNS) function via the gut-brain axis. Growing evidence suggests that gut dysbiosis not only precedes and worsens stroke severity but is also induced by stroke itself, establishing a bidirectional and self-reinforcing pathological loop. Microbiota-derived metabolites, including short-chain fatty acids and tryptophan derivatives, modulate the activation states of microglial and border-associated macrophage (BAMs), thereby shaping neuroinflammatory responses and tissue repair mechanisms. Although microglia have been extensively studied in this context, the role of BAMs-particularly perivascular-macrophages remains comparatively underexplored, despite their critical involvement in maintaining blood-brain barrier (BBB) integrity and immune surveillance. In addition, dietary patterns strongly influence microbiota composition and, consequently, immune responses within the CNS. Collectively, these findings position gut microbiota as a dynamic regulator of brain-resident immune cells in stroke and highlight diet- and microbiota-targeted interventions as promising therapeutic strategies.

## Introduction

Gut microbiota regulates many different physiological functions and gut dysbiosis can drive a plethora of diseases (Wang et al., 2025; Kaltsas et al., 2025; Blok et al., 2025; Sun T. et al., 2025). Its profile may be affected by several factors, such as gestational age, mode of delivery, maternal diet, age, antibiotic usage, diet, lifestyle or environmental exposure, among others (Van Ameringen et al., 2019). One of the most studied communication pathways involving the microbiota links it to the central nervous system (CNS) and is known as the gut-brain axis, which has been described as a crucial player in neuroimmune and neurodegenerative conditions (Tremlett et al., 2017; Elkins et al., 2024; Dalile et al., 2019; Jangi et al., 2016). Through this mechanism, microbiota metabolites influence neurodevelopment, behaviour, adult neurogenesis or the CNS immune responses (Jangi et al., 2016; Hasan Mohajeri et al., 2018; Ronan et al., 2020; Seguella et al., 2025; Guzzetta et al., 2022). Interestingly, recent studies have linked the dysfunction of the gut microbiota to the modulation of neuroinflammatory processes (Erny et al., 2021; Che et al., 2024).

Neuroinflammation is one of the main hallmarks of ischemic stroke, a condition marked by cell death and by the production of proinflammatory markers (Gong et al., 2024). Stroke, and ischemic stroke in particular, is a leading cause of both mortality and long-term disability, impacting a substantial fraction of the population and increasing the risk of neurodegenerative diseases (Johnson et al., 2019; Feigin et al., 2018; Garcia-Alloza et al., 2011). The inflammatory process implicated in brain ischemia heavily influences its outcome, as this disease usually entails a rapid loss of blood-brain barrier (BBB) integrity, followed by a migration of circulating immune cells to the ischemic brain region (DeLong et al., 2022; Planas, 2018). These cells communicate with CNS resident immune cells through cytokine secretion, thereby regulating their activity via pathways that remain incompletely understood and may exert both neuroprotective and detrimental effects on brain repair (Xie et al., 2024; Jayaraj et al., 2019). Inflammatory responses are implicated not only in stroke, but also in many other CNS pathologies such as multiple sclerosis and CNS infections (Kierdorf et al., 2019).

The unfolding of these responses largely relies on the capability of the CNS resident immune cells, such as border-associated macrophage (BAMs) and microglia (Goldmann et al., 2016). Although BAMs share several surface markers and transcriptional features with microglia, each population expresses a distinct set of markers that are largely restricted to one cell type, enabling their reliable identification and discrimination within the CNS (Kierdorf et al., 2019; Goldmann et al., 2016; Mrdjen et al., 2018; Hickman et al., 2013). BAMs encompass all non-parenchymal CNS macrophages and are classified into meningeal, choroid plexus and perivascular macrophages (PVMs), according to their anatomical location within the brain (Kierdorf et al., 2019; Goldmann et al., 2016). They play essential roles in maintaining brain homeostasis, facilitating perivascular and meningeal drainage, and orchestrating immune surveillance and inflammatory responses in the CNS (Yang et al., 2019; Park et al., 2017; Zheng et al., 2022; Henning et al., 2009; Wan et al., 2021). Within this context, microglia not only maintain CNS homeostasis by continuously sensing and modulating the activity of surrounding cells under steady-state conditions, but also serve as key effectors in inflammatory and injury responses (Nimmerjahn et al., 1979; Guzmán-Ruíz et al., 2024).

Given that the activity of these immune cells critically shapes stroke outcomes, and that their interplay is strongly influenced by gut microbiota-derived signals, understanding how these components interact is essential for unravelling the mechanisms governing the gut-brain axis.

## Gut microbiota and neuroinflammation in the context of stroke

The hypothesis of interaction between the gut and the brain was first proposed around 50 years ago, mainly based on the coincidence of certain peptides in both locations (Shulkes and Hardy, 1979). This idea evolved further over the years, until this pathway was broadened to include the gut microbiota (Bercik, 2011; Sudo et al., 2004). In fact, numerous studies investigating the gut–brain axis have demonstrated that this communication is bidirectional and operates through multiple complementary pathways, including direct neural circuits such as the vagus nerve, neuroendocrine mechanisms involving the hypothalamic-pituitary-adrenal axis, and broader endocrine signalling networks (Sudo et al., 2004; Ichiki et al., 2022; Lin et al., 2022). Within this framework, microbial metabolites, particularly short-chain fatty acids (SCFAs), have emerged as key mediators capable of entering the circulation and modulating CNS function, including attenuation of neuroinflammation following ischemic stroke (Lee et al., 2020) (see Table 1).

**TABLE 1:** Bidirectional interactions between gut microbiota and the brain in ischemic stroke.

Direction	Trigger/Event	Mediators	CNS/Gut effect	Impact on stroke outcome
Gut → Brain (protective)	Healthy microbiota	SCFAs (e.g., butyrate)	Reduced neuroinflammation; improved BBB integrity	↓ Infarct size; improved recovery
Gut → Brain (deleterious)	Dysbiosis	LPS, proinflammatory cytokines	Microglial activation; increased BBB permeability	↑ Neuroinflammation; worse outcome
Brain → Gut	Ischemic stroke	Autonomic dysfunction; systemic inflammation	Reduced microbial diversity; impaired intestinal barrier	Sustained systemic inflammation
Intervention	Microbiota restoration	FMT/recolonization	Immune rebalancing	Improved neurological recovery

Bidirectional interactions between the gut microbiota and CNS influence stroke outcome. A healthy microbiota produces SCFAs that preserve BBB integrity and limit neuroinflammation, whereas dysbiosis promotes pro-inflammatory mediators (e.g., LPS, cytokines) that exacerbate brain injury. Conversely, stroke-induced autonomic and inflammatory signals disrupt gut homeostasis, further amplifying systemic inflammation. Microbiota-targeted interventions can restore immune balance and improve recovery.

Ischemic stroke is characterized by an interruption of blood flow to a defined brain region, resulting in deprivation of oxygen and glucose (Kuriakose and Xiao, 2020). This metabolic failure rapidly activates anaerobic pathways, disrupts cellular homeostasis, and initiates cascades that culminate in excitotoxicity and cell death (Suh et al., 2008; Dirnagl et al., 1999). The ensuing release of proinflammatory cytokines and danger-associated molecular patterns drives a robust inflammatory response that mobilizes central nervous system (CNS) immune effectors (Candelario-Jalil et al., 2022). Importantly, post-stroke inflammation exerts both protective and detrimental effects, complicating therapeutic strategies aimed at modulating immune responses (Shi et al., 2021; Ma et al., 2017). Although several approaches, including immunomodulators, neuroprotective agents, and stem cell therapies, have shown promise, successful clinical translation remains limited (Haupt et al., 2023; Cao et al., 2023). In this context, targeting the gut-brain axis has emerged as a compelling alternative strategy to influence neuroinflammation and stroke outcome.

Interestingly, stroke itself profoundly alters the gut microbiota. Post-stroke dysbiosis is characterized by reduced microbial diversity and impaired intestinal barrier function (Singh et al., 2016). This altered microbial state is associated with increased systemic proinflammatory signalling and worse neurological outcomes (see Table 1). Notably, restoration of a healthy microbiota composition prior to stroke leads to exacerbated outcomes and altered immune cell profiles (Winek et al., 2016; Singh et al., 2018). Together, these findings highlight a dynamic and reciprocal relationship: the gut microbiota shapes neuroinflammatory responses after stroke, while stroke-induced systemic changes disrupt intestinal homeostasis, potentially amplifying inflammation through a self-reinforcing gut-brain loop.

Remarkably, an increase in metabolites associated with gut dysbiosis reportedly correlates with impairments in neurogenesis through the induction of inflammation and oxidative stress, affecting the repair capacity of the tissue upon injury (Zhang et al., 2022; Sarubbo et al., 2022). Furthermore, a disturbed microbiota correlates with defective myelination and altered oligodendrocyte maturation, which may potentially impact the regulation of neuron growth and repair, as well as the modulation of the inflammatory environment (Loh et al., 2024).

In mice, stroke-induced gut dysbiosis is established as soon as 3h post-stroke, quickly becoming more pronounced in the following 21h and then slowly recovering during the next 6 days, in a trend that is similarly spotted in humans, though more prolonged over time (Xu et al., 2021). This post-stroke dysbiotic state is characterized primarily by reduced microbial species diversity and evenness, while overall phylogenetic diversity appears to be relatively preserved (Yoshimura et al., 2025) (see Table 1). Mechanistically, intestinal ischemia has been identified as a downstream consequence of cerebral ischemia, contributing to elevated luminal nitrate levels that favour the expansion of facultative anaerobes such as *Enterobacteriaceae*. This bloom is associated with heightened inflammatory responses and increased infarct volume, suggesting that stroke-induced alterations in the gut microenvironment actively exacerbate brain injury (Xu et al., 2021).

Altogether, the relationship between stroke and the gut microbiota is bidirectional and self-reinforcing. While a healthy microbiota can attenuate neuroinflammation, stroke itself induces dysbiosis that may amplify systemic and central immune responses (see Table 1).

Interestingly, different bacterial families can influence post-stroke recovery in markedly different ways (Table 2). For instance, a reduced abundance of *Bacteroidetes S24-7* alongside increased levels of *Verrucomicrobiaceae*, *Lactobacillaceae, Burkholderiaceae,* and *Streptococcaceae* is associated with neuroprotection, reducing infarct volume and enhancing long-term mobility and exploratory behaviour (Benakis et al., 2020). Therefore, post-stroke microbiota repopulation is a promising avenue for addressing the issues associated with gut dysbiosis. One such strategy is faecal microbiota transplantation (FMT) (see Table 1), which involves transferring gut bacteria from a healthy donor (Vendrik et al., 2020). Supporting this, Lee et al. showed that aged, stroke-affected mice receiving FMT from young donors exhibited improved behavioural performance, strengthened gut barrier integrity, and lower levels of proinflammatory markers in the brain (Lee et al., 2020).

**TABLE 2:** Gut microbiota bacterial family impacts on stroke pathology and outcomes.

Bacteria family	Impact	Effect on stroke	Studied model	References
*Verrucomicrobiaceae*	Mixed findings	Its increased presence correlates with lower stroke damage, but other articles have raised the possibility that they may be potentially pathogenic	Mice	Benakis et al. (2020), Bao et al. (2024), Chidambaram et al. (2022)
*Lactobacillaceae*	Associated with beneficial outcomes	Lactobacillaceae abundance correlates with a lower stroke damage, being involved in post-stroke gut repair and brain anti-inflammatory mechanisms	Mice Rats	Sun T et al. (2025), Benakis et al. (2020), Chen R et al. (2019)
*Burkholderiaceae*	Associated with beneficial outcomes	Its higher abundance is tied to a reduction in post-stroke damage	Mice	Benakis et al. (2020)
*Streptococcaceae*	Associated with beneficial outcomes	Its increased presence correlates with a lower stroke damage	Mice	Benakis et al. (2020)
*Lachnospiraceae*	Associated with beneficial outcomes	This SCFA-producing family correlates with a marked reduction in post-stroke disability	Humans Mice	Chen et al. (2025), Qu et al. (2024)
*Ruminococcaceae*	Associated with beneficial outcomes	A higher abundance of SCFA-producing *Ruminococcaceae* predicts lower proinflammatory cytokine levels and lower post-stroke cognitive impairment	Humans	Jeng et al. (2025), Li et al. (2019)
*Peptostreptococcaceae*	Mixed findings	This family has been linked to depressive symptoms after stroke, but it is also associated with a favourable functional outcome	Humans Rats	Qu et al. (2024), Cai et al. (2023)
*Enterobacteriaceae*	Associated with adverse outcomes	Higher levels of lipopolysaccharide tied to the overgrowth of these bacteria correlate with aggravated brain infarction and systemic inflammation, as well as cognitive and motor impairments after stroke	Humans	Xu et al. (2021), Qu et al. (2024), Ling et al. (2020a)
*Bacteroidaceae*	Associated with adverse outcomes	A *Bacteroidaceae*-enriched microbiota is connected to higher stroke severity and proinflammatory markers	Humans Mice	Jeng et al. (2025), Xia et al. (2019)
*Clostridiaceae*	Inconsistent/controversial evidence	It was shown to correlate positively with proinflammatory markers, though an alternative study pointed to a link with decreased stroke risk	Human	Jeng et al. (2025), Zhang R et al. (2025)
*Streptococcaceae*	Associated with adverse outcomes	Its abundance is associated with the occurrence of post-stroke cognitive deficits	Humans	Ling et al. (2020b)
*Oxalobacteraceae*	Associated with beneficial outcomes	*C*orrelates positively with improvements in motor function	Humans	Qu et al. (2024)

Summary of bacterial families implicated in stroke, highlighting their beneficial, adverse, or context-dependent effects across experimental models and humans. Categories summarize reported associations and experimental findings across studies and should not be interpreted as definitive evidence of causality.

In line with these findings, it was also outlined that pre-stroke gut colonization with *Akkermansia muciniphila*, a member of the *Verrucomicrobiaceae* family, improves locomotion and mitigates brain infarct volume and cell death, through the activation of anti-inflammatory pathways (Ndongo et al., 2022; Li et al., 2024). Another study investigated the vagus nerve as a key communication pathway between the gut and the brain under conditions of transient ischemia and a high-fat diet. The researchers found that cutting the vagus nerve (vagotomy) led to reduced levels of *Akkermansia* in both ischemic and non-ischemic conditions, and that both vagotomy and arterial occlusion were associated with elevated inflammatory markers (Zhang et al., 2024). These findings, together with evidence that vagus nerve stimulation can alleviate the effects of brain ischemia, including neuroinflammation, further support the hypothesis that the gut microbiota plays a critical role in stroke outcomes, likely through a mechanism in which the vagus nerve serves as a key bridge between the gut and the brain (Jiang et al., 2014).

Nonetheless, microbiota-mediated modulation of brain function also occurs through molecular signalling driven by bacterial metabolites, such as SCFAs and tryptophan derivatives (Honarpisheh et al., 2022). SCFAs have been shown to promote the proliferation of neural progenitor niches and support neuronal maturation after ischemia, likely through the suppression of inflammatory pathways (Jaworska et al., 2019; Hasan et al., 2013).

Tryptophan metabolites, in turn, can act as ligands for the aryl hydrocarbon receptor (AHR), and their marked depletion in circulation following stroke indicates microbiota dysregulation, especially given that the bacterial populations most responsible for tryptophan metabolism are diminished in this condition (Peesh et al., 2025). This pathway is crucial in this pathology, since AHR modulation is tied to lower neurogenesis and increased neural damage (Cuartero et al., 2014; Chen WC. et al., 2019). Importantly, reintroducing these bacteria-derived metabolites into germ-free mice elicited anti-inflammatory effects and reduced neurological deficits, highlighting their potential therapeutic role (Peesh et al., 2025).

So far, early clinical studies have provided preliminary support for this model, suggesting that ischemia disrupts the gut microbiota, and that these alterations may subsequently influence brain inflammation and overall lesion outcomes (Xia et al., 2019; Zhang et al., 2023; Wu et al., 2025).

Given these links between the microbiota and immune and inflammatory responses following ischemic stroke, it is crucial to understand how the cells responsible for these processes are affected by the condition.

## Brain-resident immune cells and their responses to stroke

### Microglia

Stroke onset rapidly induces neuroinflammation, brain oedema, and oxidative stress, particularly following vessel occlusion, which markedly increases reactive oxygen species (ROS) production and damages endothelial cells forming the blood-brain barrier (BBB), thereby compromising its integrity (Candelario-Jalil et al., 2022). This disruption is further exacerbated by the rapid proinflammatory activation of resident microglia, which increases BBB permeability and vascular leakage (Lipton, 1999).

In fact, after an ischemic injury, microglia detect the molecular signature of the dying cells and respond to it by releasing proinflammatory factors such as IL-1, IL-6, TNFα, COX-2, or NOS1 (Dhungana et al., 2017). Although microglia may transiently express anti-inflammatory markers and infiltrate the ischemic core within the first 24 h post-stroke, they predominantly adopt a proliferative and proinflammatory phenotype in the peri-infarct region (Perego et al., 2011; Walberer et al., 2010). This shift in favour of developing a proinflammatory microglial state is typical of a progression towards subacute and chronic stroke (Hu et al., 2012; Lalancette-Hébert et al., 2007). Pro- or anti-inflammatory microglial polarisation has also been tied to a reduction or a boost in post-stroke neural stem cell-mediated repair, respectively (Nath et al., 2024; Zhang Q. et al., 2025).

Consequently, the microglial activation state is fundamentally related to the dynamics of the surrounding tissue, triggering and/or modulating the response of other central nervous system (CNS) cells to ischemia and *vice versa*, thereby significantly influencing the extent of tissue damage (Xu et al., 2020; Inose et al., 2015).

Nevertheless, microglia exhibit a dual role. IL-13 administration has been shown to improve outcomes in mouse stroke models by promoting anti-inflammatory markers in peri-ischemic microglia and reducing cell death (Szalay et al., 2016). Microglial-derived TNF may contribute to neuroprotection and fine-tuning of inflammation (Clausen et al., 2016; Lambertsen et al., 2012), and microglial ablation worsens neuronal excitotoxicity (Szalay et al., 2016). However, inhibition of microglial activity has also been reported to reduce post-stroke BBB dysfunction (Yenari et al., 2006).

This damage to the BBB, typical of ischemic injury, results in the infiltration of circulating monocytes, which populate the infarcted core and may participate in some tissue repair efforts shortly after the lesion onset (Miró-Mur et al., 2016). So far, the exact mechanisms triggered by this infiltration are not well characterised, therefore it is widely debated whether the effects of monocyte recruitment as a result of BBB disruption are a net positive for stroke recovery (Blank-Stein and Mass, 2023). As such, this topic is of particular interest in the field, since a broader and deeper understanding of these processes is likely to contribute to differentiating the dynamics of resident and infiltrating immune cells, allowing a more targeted approach in researching and treating this pathology.

For that reason, it is fundamental to take a closer look at the effects of ischemia on border-associated macrophages (BAMs), the macrophages tasked with maintaining the function and structure of this barrier interface.

### BAMs

BAMs are broadly classified by anatomical niche into meningeal, choroid plexus and perivascular macrophages (PVM), which show partially specialized functions across brain border compartments. Meningeal and PVMs primarily support vascular and glymphatic homeostasis through debris clearance, extracellular matrix remodelling, and regulation of CSF flow, whereas choroid plexus macrophages contribute more to CSF interface surveillance, barrier regulation, and modulation of epithelial responses. However, these populations cannot be clearly separated due to overlapping transcriptional profiles, shared markers, and functional plasticity, as well as continuous anatomical transitions between compartments. Additional complexity arises from monocyte-derived macrophages infiltrating during inflammation and adopting BAM-like states, while current dissection and genetic tools lack sufficient resolution to isolate niche-specific subsets without contamination (Sun and Jiang, 2024; Vara-Pérez and Movahedi, 2025).

Reportedly, BAMs mediate an increase in BBB permeability to facilitate the infiltration of circulating granulocytes in the acute phase of stroke, acquiring a predominantly inflammatory phenotype and presumably migrating from the periphery towards the parenchyma, though this hypothesis still requires more robust confirmation (Pedragosa et al., 2018; Rajan et al., 2020; Yu et al., 2026). The infiltrating bone marrow-derived macrophages show a modified transcriptomic profile, hinting at convergence toward an expression pattern similar to that shown by BAMs (Rajan et al., 2020).

The precise contribution of BAMs, particularly PVMs, to stroke outcomes remains controversial, with studies suggesting both beneficial and detrimental effects (Pedragosa et al., 2018; Rajan et al., 2020; Yu et al., 2026; Levard et al., 2024). These discrepancies may be explained by differences in age, stroke severity, or the marked heterogeneity within BAM populations (Yu et al., 2026; Bijnen et al., 2025). As stroke progresses beyond the acute stage and the inflammatory environment intensifies, BAMs upregulate genes associated with migration and proliferation, downregulate antigen-presentation pathways, and increase proinflammatory cytokine expression, changes that may reflect impaired function, although they also appear to contribute to vascular integrity and inflammation control during recovery (Rajan et al., 2020; Yu et al., 2026). Alternatively, it is well known that these macrophages comprise a wide range of heterogeneous populations, opening the possibility that BAMs might not act as a single unit in response to stroke, which necessitates a more detailed characterisation of these populations. BAMs are highly plastic cells whose impact depends on timing and context. They are acutely damaging by promoting vascular disruption and inflammation, subacutely heterogeneous with both reparative and inflammatory roles, and chronically capable of either supporting repair or sustaining dysfunction, depending on their long-term state and aging context (Pedragosa et al., 2018; Rajan et al., 2020; Yu et al., 2026; Levard et al., 2024).

Therapeutically, modulation of brain-resident immune cells has been explored, particularly by promoting gene expression profiles associated with an M2-like phenotype, primarily in microglia, though similar strategies in BAMs require further investigation (Gerganova et al., 2022). Alternative approaches, such as controlled depletion and repopulation of macrophages, have been proposed to preserve regenerative functions while limiting harmful effects (Gerganova et al., 2022).

Moreover, BAMs, especially PVMs, influence not only stroke response but also stroke risk by contributing to neurovascular dysfunction in hypertension, the major risk factor for stroke (Faraco et al., 2016). A deeper exploration of factors that modulate both microglial and BAM activity, including the gut microbiota, may therefore provide new avenues for mitigating ischemic brain damage.

## Gut microbiota interactions with brain-resident immune cells

Recognising that both the gut microbiota and brain-resident immune cells significantly modulate stroke outcomes, a regulatory pathway integrating the gut-brain axis emerges as a compelling area of investigation. Emerging evidence suggests that microglia and border-associated macrophages (BAMs) may be influenced by microbiota integrity, although direct evidence for BAM-specific regulation remains limited. Distinct BAM subsets appear to respond differentially to gut microbial disruptions (Sankowski et al., 2021; Erny et al., 2015), and studies in germ-free mice have reported reduced expression of M1-associated proteins in BAMs compared with conventionally colonised controls (Hove et al., 2019), supporting a potential link between microbiota status and BAM phenotype. However, much of the current understanding is based on associative findings or extrapolation from microglial research. Moreover, both microglial and BAM activation are influenced by inflammasome signalling pathways, which are themselves modulated by the gut microbiota (Wong et al., 2016; Voet et al., 2018), further suggesting possible indirect mechanisms through which microbial communities may shape these immune populations.

Gut dysbiosis has consistently been associated with a shift in microglial polarisation towards a proinflammatory phenotype. For instance, animals with disrupted microbiota exhibit enhanced microglial activation, synaptic deficiencies, and cognitive impairment (Krishnapriya et al., 2025) This effect appears to be sex-dependent and can originate as early as prenatal development, where microbiome alterations are linked to abnormal microglial maturation (Thion et al., 2018). Mechanistically, recent evidence suggests that circulating peptidoglycan fragments derived from gut bacteria may directly regulate microglial activation states (Spielbauer et al., 2024). Additionally, dysbiosis promotes lipid droplet accumulation in microglia, impairing their phagocytic capacity and exacerbating inflammatory responses, a phenomenon that may plausibly extend to BAMs, although this remains to be experimentally confirmed (Hasavci and Blank, 2022).

Microbial metabolites further illustrate this regulatory axis. Short-chain fatty acids (SCFAs), particularly sodium butyrate, have been shown to target microglia and attenuate neuronal damage in neurodegenerative disorders such as Parkinson disease (PD) and Alzheimer disease (AD), conditions frequently accompanied by gut dysbiosis (Lv et al., 2024; Xu et al., 2025; Liu et al., 2024). In AD, microbiota alterations also influence BAMs, especially perivascular macrophages (PVM), modulating their capacity to clear β-amyloid and thereby affecting disease severity (Sankowski et al., 2021; Silvin et al., 2023). In both disorders, supplementation with specific bacterial species mitigates inflammatory phenotypes and improves outcomes (Lv et al., 2024; Xu et al., 2025; Liu et al., 2024).

Similar mechanisms have been observed in the context of stroke. Faecal microbiota transplantation (FMT) from young mice into aged stroked mice reduces microglial activation, reinforcing the regulatory role of microbiota (Lee et al., 2020). Administration of *Akkermansia muciniphila* in stroked mice promotes a shift towards an M2-like, anti-inflammatory microglial phenotype (Li et al., 2024), while sodium butyrate supplementation attenuates M1-like polarisation and neuronal death following cerebral ischemia (Sun J. et al., 2025; Patnala et al., 2016). Furthermore, greater microbial diversity has been associated with reduced severity of stroke sequelae, including post-stroke depression (Chen et al., 2026; Jiang et al., 2025). Moreover, novel studies have identified molecules with therapeutic potential in stroke, having a modulatory effect on the diversity and composition of the microbiota, thereby stimulating a neuroprotective microglial state (Chen et al., 2025; Huang et al., 2024) (see Table 3).

**TABLE 3:** Microbiota-mediated modulatory effects of different molecules with impact on stroke and immune responses.

Molecules	Effect on microbiota and immune system	Stroke outcome	References
Short-course of Broad-spectrum antibiotics (microbiome remodelling approach)	Alter gut microbiota composition and reduce microbial diversity Modifies immune responses by lowering activation of microglia/macrophages, decreasing pro-inflammatory cytokines, and reducing infiltration of peripheral immune cells into the brain. However, it may also induce gut dysbiosis and reduce SCFA levels	Reduced neuroinflammation, smaller brain lesion volume, and decreased neuronal cell death after traumatic brain injury (experimental model)	Flinn et al. (2026)
SCFAs (acetate, propionate, butyrate)	Produced by bacterial fermentation of dietary fibre. They regulate immune responses by promoting Treg differentiation, reducing pro-inflammatory cytokines, strengthening the intestinal barrier, and modulating microglial maturation and activity	Generally neuroprotective: associated with reduced neuroinflammation, improved immune regulation, and better neurological recovery after ischemic stroke	Chen et al. (2024)
Polyphenols	Polyphenol-rich foods have been shown to increase the presence of SCFA-producing bacteria in the gut, thus promoting the production of these compound and its downstream anti-inflammatory effects	Linked to SCFA-mediated neuroinflammation decrease and better overall stroke outcomes	Gates et al. (2022)
Lipopolysaccharide (LPS)	A component of the cell wall of some bacteria, LPS can enter the bloodstream in cases when the intestinal permeability is heightened. It elicits macrophage M1-like activation, prompting inflammation, and triggering pathways that can lead to oxidative stress and cell death	Positively correlates with neuroinflammation and BBB disruption, thus leading to worse overall stroke outcome	Guo et al. (2025)
Tryptophan-derived aryl hydrocarbon receptor (AHR) ligands	AHR ligands are produced through the bacterial metabolism of tryptophan. The activation of microglial AHR inhibits the NF-κB pathway, lowering inflammatory cytokine levels	The activation of AHR through these ligands can reduce neuroinflammation, promoting recovery from the injury	Guo et al. (2025), Ouyang et al. (2025)
Phenylacetylglutamine (PAGln)	Metabolite resulting from gut microbiota activity. There is no clear pathway linking PAGln to inflammation, but its systemic diffusion has been shown to be involved in the activation of inflammatory pathways	Higher plasma PAGln levels are linked to the occurrence of ischemic stroke, mainly through the promotion of thrombus formation	Krishnamoorthy et al. (2024)
Atorvastatin	Atorvastatin is a drug that is able to increase the relative abundance of beneficial, SCFA-producing bacteria in the gut. Increased SCFA production is associated with neuroprotection	Improvements seen in neuroinflammation and neurobehavioural deficits	Chen et al. (2025), Vieira-Silva et al. (2020)
*Acorus tatarinowi* (AT) oils	These plant extracts have been shown to help maintain a balanced gut microbiota, consequently decreasing proinflammatory cytokine levels and inducing an M2-like microglial polarisation	Fosters neuroprotection and the recovery of neurological function, reducing the deleterious effects of ischemic stroke	Huang et al. (2024)
TMAO	Microbiota-dependent metabolic product of the breakdown of dairy, eggs, fish and meat. Promotes the disruption of the BBB and a neuroinflammatory environment	Increases stroke severity and worsens stroke outcomes by fostering inflammation. It has also been associated with a higher risk of stroke, through thrombosis and atherogenesis	Guo et al. (2025), Peh et al. (2022)

Overview of key microbiota-derived metabolites and interventions that shape immune responses and stroke pathology. Anti-inflammatory mediators such as SCFAs, polyphenols, and AHR ligands generally promote immune regulation, preserve barrier integrity, and improve neurological recovery. In contrast, pro-inflammatory or pro-thrombotic molecules (e.g., LPS, TMAO, PAGln) are associated with enhanced neuroinflammation, BBB disruption, and worse stroke outcomes. Microbiota-modulating interventions, including antibiotics, statins, and plant-derived compounds, exert context-dependent effects by reshaping microbial composition and downstream immune responses.

Beyond stroke and neurodegeneration, the microbiota-immune axis may also operate in other inflammatory CNS conditions. In HIV infection, gut dysbiosis contributes to systemic and cerebral inflammation, potentially regulating brain macrophage activity and shaping disease progression (Torices et al., 2023). Given that HIV is associated with increased stroke incidence and poorer outcomes, microbiota-driven inflammasome activation may represent a mechanistic link predicting greater neuronal loss and cognitive decline in affected individuals (Torices et al., 2023).

Despite the accumulating evidence linking the gut microbiota to microglial regulation, studies specifically addressing its impact on BAM function, particularly in the context of cerebral ischemia, remain scarce. This represents a significant gap in the literature, as deeper characterisation of microbiota-driven modulation of BAMs may provide critical insights into how the gut-brain axis influences stroke pathophysiology and recovery.

## Gut-brain axis macrophages quorum sensing

Within the intestine, lamina propria macrophages represent the largest and most dynamic macrophage population, forming a dense network beneath the epithelium and around the mucosal vasculature (Honda et al., 2020; Viola and Boeckxstaens, 2021). Constantly exposed to luminal antigens and the microbiota, they exist in a state of controlled physiological inflammation and require continuous replenishment from circulating monocytes, in contrast to deeper gut-wall macrophages that partially self-maintain (Viola and Boeckxstaens, 2021). Notably, lamina propria macrophages exhibit a highly regular spatial distribution and proliferate when niches become available, suggesting that they can sense local cell density and adjust their numbers accordingly (Viola and Boeckxstaens, 2021). This population-level regulation-potentially mediated by trophic factors such as Colony-stimulating factor 1 (CSF1), which is consumed by resident macrophages and accumulates upon cell loss to trigger proliferation and monocyte recruitment-resembles a form of quorum sensing (Viola and Boeckxstaens, 2021).

A conceptual parallel can be drawn to brain-associated macrophages (BAMs), which line vascular and meningeal interfaces and likewise display ordered territorial organization and niche-dependent maintenance. Although separated by anatomical barriers, lamina propria macrophages and brain BAMs are exposed to shared systemic cues, including circulating cytokines, microbial metabolites such as short-chain fatty acids (SCFAs) or lipopolysaccharide, and neuroendocrine signals transmitted via the vagus nerve or hypothalamic-pituitary-adrenal axis (Park et al., 2025). When gut inflammation reaches a critical threshold, accumulated cytokines and microbial products may alter blood-brain barrier (BBB) integrity and modulate BAM and microglial activation, thereby functionally linking intestinal and cerebral immune responses.

Thus, rather than functioning as isolated populations, lamina propria macrophages and brain BAMs may participate in a bidirectional, density- and threshold-dependent communication network that echoes quorum sensing principles. While direct evidence for density-dependent inter-organ macrophage communication remains lacking, these shared regulatory principles raise the possibility that gut and brain barrier macrophages may participate in coordinated responses through common systemic signalling pathways. Further experimental studies simultaneously assessing macrophage density, activation states, and spatial organization across organs will be necessary to determine whether such interactions reflect broader quorum sensing-like mechanisms within the gut-brain axis (Viola and Boeckxstaens, 2021).

## Dietary modulation of the gut-brain axis

Given the sensitivity of the gut microbiota to environmental inputs, diet emerges as a central regulator of the gut-brain axis. Increasing evidence suggests that dietary composition can reshape microbial architecture, thereby influencing brain-resident immune cell activity and ultimately modulating stroke risk and neurological outcomes.

Pro-inflammatory dietary patterns, particularly the Western diet, characterised by high levels of refined carbohydrates and saturated fats, have been shown to disrupt microbiota composition and impair cognition (Crain et al., 2025). High-fat diet (HFD) consumption is strongly associated with gut dysbiosis, increased intestinal permeability, and heightened macrophage reactivity, culminating in systemic and neuroinflammation (Mukherjee et al., 2023; Roy et al., 2024). Within the central nervous system (CNS), HFD influences both microglia and border-associated macrophages (BAMs), promoting blood-brain barrier (BBB) damage and angiopathy while enhancing proinflammatory activation states (Valdearcos et al., 2017; Mendes and Velloso, 2022). Interestingly, BAMs may also mount a compensatory neuroprotective response under HFD conditions, suggesting functional plasticity that warrants deeper mechanistic investigation (Jais et al., 2016). The effect of this dietary model on BAMs is seemingly intensified if the exposure happens earlier in the individual’s life, during development or lactation, with an increased presence of CD206, a protein linked to phagocytosis (Zheng et al., 2022; Hawkes et al., 2015). Whereas in ageing, HFD disrupts microglial homeostasis across multiple CNS regions, amplifying proinflammatory cytokine production and promoting chronic neuroinflammation (Evans et al., 2024; Spencer et al., 2019). Collectively, these findings position HFD as a potent driver of gut-brain communication toward a proinflammatory state associated with cognitive impairment and neurodegenerative disease risk (Lin et al., 2023; Cope et al., 2018).

Similarly, negative repercussions have been reported as a result of elevated levels of trimethylamine N-oxide (TMAO), a product of microbiota-dependent metabolic breakdown of dairy, egg, fish and meat (Peh et al., 2022). An overconsumption of these carnitine- and coline-rich products correlates with changes in microbiota composition (Koeth et al., 2013). Dysbiosis may exacerbate TMAO accumulation, and higher circulating levels correlate with increased ischemic stroke severity (Zhu et al., 2021), reinforcing the link between dietary substrates, microbial metabolism, and cerebrovascular risk.

In contrast, several dietary patterns appear to promote neuroprotection through microbiota modulation. Fibre-rich diets enhance microbial diversity and stimulate short-chain fatty acids (SCFAs) production, which exerts anti-inflammatory effects and regulates macrophage activity (Threapleton et al., 2013; Sharon et al., 2014). SCFAs, including butyrate, have demonstrated protective roles in neurodegenerative disorders and cerebral ischemia by attenuating proinflammatory macrophage and microglial responses (Hasavci and Blank, 2022; Moțățăianu et al., 2023; Megur et al., 2021). Curiously, SCFA profiles may be amplified through the intake of salmon protein, a nutrient which not only fosters neuroprotection, but also promotes a healthy microbiota (Guan et al., 2025).

Polyphenol-rich foods represent another promising avenue. Compounds found in cranberries, grapes, pomegranate, and *Ugni molinae* (Anhê et al., 2016; Yang et al., 2021; Cisternas-Olmedo et al., 2025) promote the growth of beneficial bacteria such as *Akkermansia muciniphila*, suppress microglial proinflammatory polarisation, and confer neuroprotective effects in conditions including ischemic stroke (Gates et al., 2022), Parkinson disease (PD) (Gates et al., 2022), Alzheimer´s disease (AD) (Gates et al., 2022) and Huntington’s disease (Anhê et al., 2016; Yang et al., 2021; Cisternas-Olmedo et al., 2025) Soy-derived isoflavones have similarly been associated with mitigation of HFD-induced dysbiosis and reduced hypothalamic inflammation (Pang et al., 2020; Nakashima et al., 2025; Tura and Lemma, 2019). The abundance of *Akkermansia* further correlates negatively with circulating proinflammatory saturated fatty acids, suggesting a regulatory role in systemic inflammatory tone (Rodríguez-Carrio et al., 2017).

Beyond polyphenols and fibre, omega-3 unsaturated fatty acids also contribute to microbiota-mediated neuroprotection. As a matter of fact, omega-3-rich fish oil consumption was observed to contribute to promote a healthy gut microbiota, resulting in improved cognition and social behaviour (Jin et al., 2020). Unlike saturated or non-omega-3 unsaturated fatty acids, omega-3 levels do not negatively correlate with *Akkermansia* abundance, suggesting a microbiota-friendly lipid profile (Rodríguez-Carrio et al., 2017). Fermented foods such as kefir further exemplify microbiota-targeted dietary strategies, enhancing beneficial bacterial populations and improving inflammatory balance, neurotransmission, and cognitive performance (Kim et al., 2019; Wouw et al., 2020) Similarly, seaweed supplementation may restore microbiota-driven amino acid metabolism and reinforce BBB integrity, contributing to motor and cognitive improvements in neurodegenerative contexts (Gates et al., 2022).

Though these findings by themselves require further studies to confirm their strength, particularly in human subjects and in a clinical context, they consolidate the concept that diet is a powerful upstream regulator of the gut-brain axis, when articulated together. Pro-inflammatory dietary patterns appear to promote dysbiosis, immune activation, and increased cerebrovascular vulnerability, whereas fibre-, polyphenol-, omega-3-, and probiotic-rich diets support microbial diversity, anti-inflammatory immune states, and neurovascular protection. Crucially, these dietary components show great overlap in the mechanisms they activate to foster neuroprotection, and the metabolites that mediate this interplay, such as SCFAs, may reportedly engage similar pathways in humans (Peh et al., 2022; Tan et al., 2021). Therefore, it is expected that the same effect may be seen in stroke patients who incorporate these elements in their diet, though this particular hypothesis must be verified in future experimental works. Consequently, dietary modulation represents a plausible and accessible strategy for influencing microbiota–immune interactions and potentially mitigating stroke risk and severity.

## Conclusion

**FIGURE 1:**
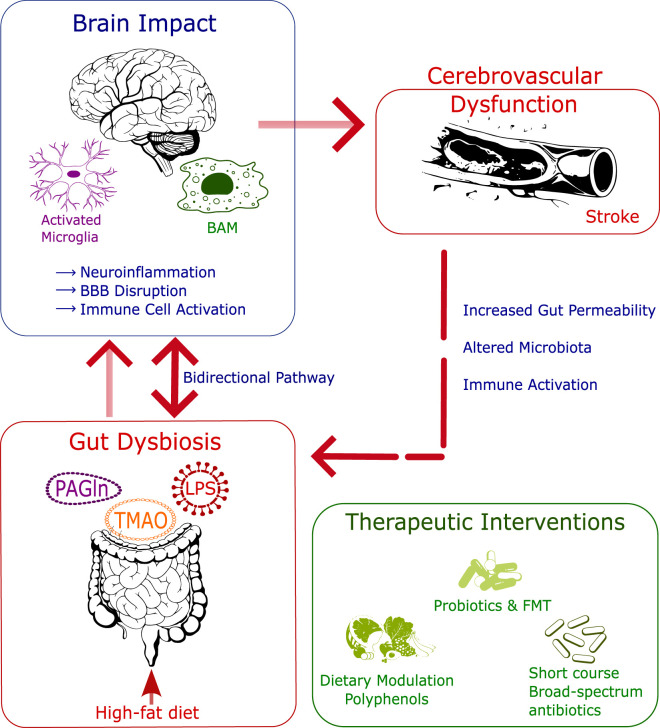
Brain-gut axis interactions in stroke and therapeutic modulation. Schematic representation of the bidirectional communication between the brain and gut in the context of cerebrovascular dysfunction and stroke. At the CNS level, activation of microglia and brain-associated macrophages (BAMs) contributes to neuroinflammation, blood-brain barrier (BBB) disruption, and immune cell activation, promoting stroke pathology. Stroke-induced cerebrovascular dysfunction further impacts the gut via autonomic and inflammatory pathways, leading to increased intestinal permeability, altered microbiota composition, and systemic immune activation. In turn, gut dysbiosis-characterized by elevated levels of metabolites such as lipopolysaccharide (LPS), trimethylamine N-oxide (TMAO), and phenylacetylglutamine (PAGln), often associated with high-fat diets-feeds back to exacerbate neuroinflammation and brain injury. This establishes a self-reinforcing pathological loop. Therapeutic interventions, including probiotics, faecal microbiota transplantation (FMT), dietary modulation (e.g., polyphenols), and short-course antibiotics, aim to restore microbial balance and modulate immune responses, thereby mitigating stroke severity and promoting recovery.

Ischemic stroke and the gut microbiota engage in a dynamic, bidirectional interaction that critically shapes neuroinflammation and neurological outcomes (Figure 1). Although microglial responses to gut-derived signals have been increasingly characterised, and the impact of these macrophages in regulating inflammation and neurogenesis after stroke is starting to be uncovered, the contribution of BAMs remains poorly defined. This is further complicated by their heterogeneity, niche-dependent maintenance, and the difficulty of resolving distinct BAM subsets, as well as their potential coordination with peripheral macrophage populations, which together point to a more integrated and dynamic immune network than previously recognized. Defining how microbial signals shape BAM function therefore represents a critical unmet challenge in the field. Similarly, the optimal timing for microbiota-based interventions-including preventive strategies before stroke onset, as well as interventions during the acute phase or chronic recovery stage-remains unclear. This is particularly relevant given that microbiota modulation prior to disease onset may reduce baseline systemic inflammation and strengthen barrier integrity, potentially lowering stroke susceptibility, whereas post-stroke interventions may instead aim to limit secondary neuroinflammation and support repair. However, immune responses evolve substantially over time and may exert both protective and detrimental effects depending on context.

In addition, key variables such as age, sex, and comorbidities (e.g., hypertension, metabolic disease) significantly influence both gut microbiota composition and neuroimmune responses, yet their combined impact on the gut–brain axis in stroke is still insufficiently characterized. These factors likely contribute to the variability observed across experimental and clinical studies and should be systematically addressed in future work. Unfortunately, there is a distinct lack of clinical trials targeting the modulation of the bacterial populations in the gut in stroke, leaving a knowledge gap regarding the influence of these intrinsic factors, the optimal timing of intervention and potential safety concerns of this therapeutic strategy that must be addressed going forward.

Importantly, emerging therapeutic strategies, including stem cell-based regenerative approaches, may benefit from integration with microbiota-targeted interventions. Given that stem cell efficacy is closely linked to the host inflammatory environment, modulation of the gut-brain axis could enhance regenerative outcomes by promoting a more permissive immune landscape.

Overall, a deeper and more precise understanding of BAM-specific regulation, temporal dynamics of intervention-including prevention-and host-dependent variables will be essential for translating microbiota-based strategies into effective therapies for stroke.
